# Determinants of rural-urban differences in health care provider visits among women of reproductive age in the United States

**DOI:** 10.1371/journal.pone.0240700

**Published:** 2020-12-10

**Authors:** Hyunjung Lee, Ashley H. Hirai, Ching-Ching Claire Lin, John E. Snyder

**Affiliations:** 1 Oak Ridge Institute for Science and Education (ORISE), Oak Ridge, Tennessee, United States of America; 2 Office of Health Equity (OHE), Health Resources and Services Administration (HRSA), Rockville, Maryland, United States of America; 3 Maternal and Child Health Bureau (MCHB), Health Resources and Services Administration (HRSA), Rockville, Maryland, United States of America; 4 Office of Planning, Analysis, and Evaluation (OPAE), Health Resources and Services Administration (HRSA), Rockville, Maryland, United States of America; Federal University of Pelotas, BRAZIL

## Abstract

**Background:**

Rural health disparities and access gaps may contribute to higher maternal and infant morbidity and mortality. Understanding and addressing access barriers for specialty women’s health services is important in mitigating risks for adverse childbirth events. The objective of this study was to investigate rural-urban differences in health care access for women of reproductive age by examining differences in past-year provider visit rates by provider type, and quantifying the contributing factors to these findings.

**Methods and findings:**

Using a nationally-representative sample of reproductive age women (n = 37,026) from the Medical Expenditure Panel Survey (2010–2015) linked to the Area Health Resource File, rural-urban differences in past-year office visit rates with health care providers were examined. Blinder-Oaxaca decomposition analysis quantified the portion of disparities explained by individual- and county-level sociodemographic and provider supply characteristics. Overall, there were no rural-urban differences in past-year visits with women’s health providers collectively (65.0% vs 62.4%), however differences were observed by provider type. Rural women had lower past-year obstetrician-gynecologist (OB-GYN) visit rates than urban women (23.3% vs. 26.6%), and higher visit rates with family medicine physicians (24.3% vs. 20.9%) and nurse practitioners/physician assistants (NPs/PAs) (24.6% vs. 16.1%). Lower OB-GYN availability in rural versus urban counties (6.1 vs. 13.7 providers/100,000 population) explained most of the rural disadvantage in OB-GYN visit rates (83.8%), and much of the higher family physician (80.9%) and NP/PA (50.1%) visit rates. Other individual- and county-level characteristics had smaller effects on rural-urban differences.

**Conclusion:**

Although there were no overall rural-urban differences in past-year visit rates, the lower OB-GYN availability in rural areas appears to affect the types of health care providers seen by women. Whether rural women are receiving adequate specialized women’s health care services, while seeing a different cadre of providers, warrants further investigation and has particular relevance for women experiencing high-risk pregnancies and deliveries.

## Introduction

Health disparities for people living in rural areas are well-documented, with rural residents having overall higher mortality rates and lower life expectancy levels than their urban counterparts, which has been attributed to factors such as poverty, health care provider shortages, longer average distances from healthcare facilities, and rural hospital closures [1–3]. Rurality may also be an important factor specifically for maternal and infant morbidity and mortality rates. For example, rural residents have been found to have a 9 percent greater probability of severe maternal morbidity and mortality and a 6 to 20 percent higher rate of infant mortality [4, 5]. Higher rates of Medicaid reliance and uninsurance likely to contribute to rural women being more likely to delay or forgo seeking health care and, along with higher prevalence of chronic health conditions and substance use, this may impact the adverse maternal and infant outcomes observed in rural areas [4, 6, 7].

Health workforce insufficiencies and specifically more limited access to specialized obstetric providers are also believed to contribute to the higher rates of severe maternal morbidity and mortality events observed in rural areas [4]. Obstetrician-gynecologists (OB-GYNs), family physicians, and certified nurse midwives are all well-equipped to provide specialty women’s health services, including guideline-based perinatal care and obstetrical deliveries [6, 8]. Studies suggest that OB-GYNs, family physicians, and certified nurse midwives may have comparable rates for obstetric outcomes relevant for maternal morbidity and mortality, such as postoperative complications, perinatal mortality, and adverse maternal outcomes [9–12]. However, access for rural women to high-quality care by essential maternal health care providers during both pregnancy and childbirth appears to be limited, at least in part, by shortages of these providers [1]. There are disproportionate regional shortages of OB-GYNs and primary care physicians across the nation, health workforce deficiencies that are projected by the federal Health Resources and Services Administration (HRSA) to worsen in coming years [13, 14]. Provider shortages often coexist in rural communities with prevalent, adverse social determinants of health that serve to increase maternal mortality risk [6, 15]. The importance of understanding and addressing differences in women’s health care access is further underscored by rising rates of severe maternal morbidity and mortality and increasing hospital obstetric unit closures in rural areas with noted consequences for birth outcomes [4, 16].

A recent analysis showed that rural women have lower rates of having had any past-year physician visits, and also specifically any obstetrician-gynecologist visits, than their urban counterparts but did not examine non-physician visits or identify contributors [17]. Building off this recent work and that of others, this study aims to further cultivate a richer, more detailed understanding of rural health care access disparities for women of reproductive age, in order to inform ameliorative policy interventions [4, 18–20]. This study seeks to identify which individual- and county-level sociodemographic and provider supply characteristics are the key contributors to rural-urban disparities in whether women of reproductive age have had past-year office-based provider visits with the various providers relevant to women’s health care. Among these, this study specifically investigates whether observed rural disparities relate to the availability of physicians in the specialties of obstetrics-gynecology, family medicine, internal medicine, and general practice, as well as non-physicians, including certified nurse-midwives, nurse practitioners (NPs), and physician assistants (PAs) [21].

## Methods

### Data/Sample

The primary data source for this study was the Medical Expenditure Panel Survey (MEPS). Conducted by the Agency for Healthcare Research and Quality (AHRQ), MEPS is the most complete source of nationally representative survey data on U.S. health care costs, utilization, and insurance coverage. The MEPS Household Component (MEPS-HC) consists of an overlapping panel design, including 5 rounds of interviews over 2 years [22]. The survey methodology is described in detail on the AHRQ website [23]. Six years (2010–2015) of full-year, consolidated MEPS-HC data files were merged with year-matched county-level variables from HRSA’s Area Health Resource File (AHRF) for this analysis. This range of years reflected the latest available datasets for study within the period since passage and implementation of the Affordable Care Act. The study sample was restricted to women of reproductive age (n = 39,462), defined here as 18 to 44 years old, as this age group encompasses the main users of maternal health care services and accounted for 99% of births in 2018 [24]. The study was exempt from Institutional Review Board approval as it utilized a de-identified public dataset.

### Outcome

Self-reported receipt of past-year office-based visits with various health care providers were the dichotomous (yes/no) outcome measures of this study, with a focus on the practitioners caring for women of reproductive age and relevant for mitigating maternal mortality. Within MEPS-HC data, for each provider type studied, survey respondents were asked if in the prior 12 months they had “saw or talked to the provider,” defined in the survey as a telephone- or office-based visit. Only office-based visits were used for analysis, and visits for a vision exam, laser eye surgery, or well child exam were excluded to better capture in-person appointments related to a woman’s general health. Telephone-based visits were excluded from analysis as these are inconsistent with how telemedicine visits are most often characterized, which is real time interactive communication between a patient and a distant provider using both audio and video modalities [25]. Similar to a prior study, visit rates to various providers of women’s health care were examined overall, individually, and categorically to differentiate between outcomes ascribable to the role of a specific specialty or to a particular service need that can be provided by more than one occupation [21]. Birth supervisors, those who may directly attend deliveries, included OB-GYNs, family medicine physicians, and certified nurse-midwives. Primary care providers included non-subspecialist physicians in internal medicine, family medicine, and general practice, as well as NPs and PAs. Primary care providers were examined due to their important role in providing comprehensive chronic disease management before, during, and after pregnancy, and because nearly two-thirds of pregnant women report receiving care from a mix of clinicians, including OB-GYNs, family physicians, midwives, NPs, and PAs [26, 27].

### Rural/Urban classification

As commonly applied in federal policy, rural and urban residency was classified at the county level based on the Office of Management and Budget (OMB) definition of rurality, using metropolitan and micropolitan statistical areas. Rural counties included micropolitan counties (with populations of 10,000 to 50,000) or noncore counties, and urban counties included metropolitan counties (with populations of 50,000 or more) [3]. Although the OMB county definition is not as granular as alternatives based on the census tract (e.g., Rural Urban Commuting Area codes), data at the census tract level was not available and the county-level data was consistent with other covariates [3].

### Individual- and county-level characteristics

Based on prior studies of women’s health care access and health disparities, individual-level predictors were selected as covariates, including age, race, ethnicity, educational status, marital status, family income, health insurance status, family size, employment status, pregnancy status, U.S. Census region, and self-perceived poor or fair general and mental health status [1, 6, 7, 15, 21, 28]. Also, using prior studies for guidance, key county-level characteristics from the AHRF were selected, including unemployment rates and the number of obstetrics-gynecology physicians, non-subspecialist primary care physicians, nurse practitioners, and physician assistants per 100,000 population [1, 7, 16, 21].

### Analytic approach

Crude differences were calculated for past-year provider visit rate outcomes and covariates between rural and urban women. Based on these results, for each of the provider types where rural-urban differences in visit rates were observed at a level of statistical significance, linear probability regression models were developed for further investigation into the underlying factors associated with these findings, while adjusting for covariates. From these adjusted models, Blinder-Oaxaca decomposition analysis was conducted to quantify the contributions of individual and county characteristics in explaining the observed rural-urban differences in access to care [29, 30].

$${\bar{y}}_{rural\;}-{\bar{y}}_{urban}=\underset{\text{Explained}}{\underset{︸}{({\bar{x}}_{rural\;}-{\bar{x}}_{urban}){\hat{\beta }}^{*}}}+\underset{\text{Unexplained}}{\underset{︸}{{\hat{\beta }}_{rural})}}$$

In the equation above, and ${\bar{y}}_{rural}$ and ${\bar{y}}_{urban}$ represent means of the rates of health care provider visits for rural and urban residents, and ${\bar{x}}_{rural}$ and ${\bar{x}}_{urban}$ are vectors of group-specific means of the individual- and county-level characteristics studied. ${\hat{\beta }}^{*}$ is the vector of coefficients from the pooled linear probability models that reflects the average outcome difference associated with each covariate. ${\hat{\beta }}_{rural}$ is the adjusted difference in outcomes between rural and urban women that remains unexplained by covariates. The “explained” portion of the decomposition reflects the change in the mean of health care visits for rural residents, compared to urban residents, due to the covariate differences in the individual- and county-level characteristics studied. The percentage of the outcome difference explained by each covariate can be obtained by dividing the explained difference by the total difference ($\frac{({\bar{x}}_{rural}-{\bar{x}}_{urban}){\hat{\beta }}^{*}}{{\bar{y}}_{rural}-{\bar{y}}_{urban}}$). Although a linear decomposition approach was used due to the computational and interpretational parsimony of point estimates and standard errors, decomposition analysis was also conducted with the non-linear approach developed by Fairlie as a sensitivity analysis [31]. Respondents with missing values for any of the individual- and county-level variables (n = 2,436, 6.6%) were excluded from all analyses, for a final sample size of 37,026. Collinearity between model covariates was assessed using variance inflation factors, and all values across models were within an acceptable range (<2.2). All statistical analyses accounted for the complex sampling design, with weighting to represent the population of non-institutionalized U.S. reproductive age women, and were conducted using STATA 15 at the AHRQ Data Center.

## Results

### Medical provider visits by rural and urban residence

Overall, there was no rural-urban difference in having had any past-year medical provider visits or birth supervisor visits (Table 1). However, among the birth supervisors, rural women were less likely to have had an obstetrician-gynecologist visit (23.3% rural vs. 26.6% urban, p<0.05) and more likely to have had a family medicine physician visit (24.3% rural vs. 20.9% urban, p<0.05). Rural-urban differences in visit rates with certified nurse-midwives were not statistically significant (1.7% versus 0.9%, p = 0.07). Overall, rural women were more likely to have reported a past-year primary care provider visit than urban women (54.3% rural vs. 50.1% urban, p<0.05), although this was attributable to the greater rural likelihood of having had NP/PA visits (24.6% rural vs. 16.1% urban, p<0.05) since there was no meaningful rural-urban difference in visit rates with primary care physicians as a collective grouping. Thus, the lower rural rate of OB-GYN visits and the higher rural rates of visits with family medicine physicians and NPs/PAs were selected for multivariable regression and decomposition analysis.

**Table 1:** Past-year medical provider visits (%) among women age 18–44 by rural/urban residence.

	Weighted Percentages (SE)		Differences (95% CI)	*P*
	Rural (n = 4,139)	Urban (n = 32,887)		
**Any medical provider visits**^†^	65.0 (1.6)	62.4 (0.5)	2.6 (-0.6,5.8)	0.115
**Visits with birth supervisors**	42.6 (1.6)	42.1 (0.5)	0.6 (-2.9,4.0)	0.750
• **Obstetrics-gynecology physicians**	23.3 (1.4)	26.6 (0.5)	-3.3 (-6.1,-0.4)*	0.024
• **Family medicine physicians**	24.3 (1.6)	20.9 (0.5)	3.4 (0.2,6.6)*	0.037
• **Certified nurse midwives**	1.7 (0.4)	0.9 (0.1)	0.7 (-0.1,1.5)	0.070
**Visits with primary care providers**	54.3 (1.5)	50.1 (0.6)	4.2 (1.1,7.3)*	0.008
• **Primary care physicians**^‡^	40.6 (1.5)	41.9 (0.4)	-1.3 (-4.4,1.8)	0.410
• **Nurse practitioners and physician assistants**	24.6 (1.7)	16.1 (0.5)	8.5 (4.9,12.1)*	<0.001

Note: Sample size = 37,026.

* Estimate is different from the urban estimate at a level of p<0.05.

^†^ Includes family medicine physicians, general practice physicians, internal medicine physicians, obstetrics/gynecology physicians, certified nurse midwives, nurse practitioners, physician assistants.

^‡^ Includes family medicine physicians, general practice physicians, and internal medicine physicians.

### Population characteristics by rural and urban residence

Rural-urban differences were observed in a number of individual- and county-level characteristics known to typically affect access to health care providers (Table 2). Among the individual factors, rural women of reproductive age were more likely to be non-Hispanic white, unemployed, married, pregnant, and have a large family size, lower level of education and income, and poorer self-reported health status. Compared to urban women, rural women had lower per-capita county supplies of obstetrician-gynecologists (6.1 vs. 13.7 providers per 100,000 population) and NPs/PAs (59.2 vs. 79.5 providers per 100,000 population). Rural-urban differences in per-capita county supplies of family medicine physicians did not reach a level of statistical significance (27.0 vs. 28.1 providers per 100,000 population).

**Table 2:** Individual and county characteristics among women age 18–44 by rural/urban residence.

Individual and county characteristics		Weighted Means (SE)		*P*
		Rural (n = 4,139)	Urban (n = 32,887)	
**Individual-level characteristics**				
**Age (years)**		30.7 (0.2)	30.9 (0.1)	0.417
**Race/ethnicity**	White, Non-Hispanic	77.4 (2.9)*	55.1 (1.2)	<0.001
	Black, Non-Hispanic	9.1 (1.5)*	14.3 (0.8)	
	Other Race, Non-Hispanic	4.5 (1.5)*	9.8 (0.6)	
	Hispanic (all races)	9.0 (2.1)*	20.8 (1.1)	
**Education**	Less than High School	16.7 (1.0)*	14.0 (0.5)	<0.001
	High School	36.5 (1.5)*	29.3 (0.5)	
	Bachelors/Graduate Degree	46.7 (1.9)*	56.8 (0.7)	
**Married**		50.9 (1.5)*	44.7 (0.7)	0.001
**Income group**	Low Income	44.7 (1.8)*	35.3 (0.7)	<0.001
	Middle Income	34.0 (1.2)*	30.7 (0.5)	
	High Income	21.3 (1.6)*	34.0 (0.7)	
**Health insurance**		82.7 (1.6)	85.2 (0.6)	0.137
**Family size**		3.4 (0.1)*	3.3 (0.0)	0.035
**Employed**		56.8 (1.3)*	60.2 (0.6)	0.024
**Poor or fair health status**		8.4 (0.6)*	6.7 (0.3)	0.007
**Poor or fair mental health status**		5.4 (0.8)	4.5 (0.2)	0.241
**Pregnant**		14.6 (0.9)*	12.3 (0.3)	0.015
**Region**	Northeast	10.8 (3.8)*	18.3 (0.6)	<0.001
	Midwest	33.8 (3.7)*	19.1 (0.9)	
	South	41.8 (3.8)*	37.1 (1.0)	
	West	13.7 (3.3)*	25.5 (1.0)	
**County-level characteristics**				
**County unemployment rate**		8.0 (0.3)*	7.4 (0.1)	0.049
**Obstetrics-gynecology physicians per 100,000 population**		6.1 (0.6)*	13.7 (0.2)	<0.001
**Nurse practitioners and physician Assistants per 100,000 population**		59.2 (3.8)*	79.5 (1.4)	<0.001
**Family medicine physicians per 100,000 population**		27.0 (1.7)	28.1 (0.5)	0.499

Note: Sample size = 37,026.

Note:

*Estimate is different from the urban estimate at a level of p<0.05.

### Linear probability models

After adjustment for individual and county-level characteristics, the rural-urban differences in OB-GYN visit rates (-2.7%; 95% CI = -4.9,-0.5) and NP/PA visit rates (6.1%; 95% CI = 2.7,9.4) were still significant, however family medicine physician visit rates were not (-1.0%; 95% CI = -4.3, 2.3) (Table 3). Overall, most of the characteristics studied were significantly associated with at least one of the outcome measures. For example, women who were white, pregnant, employed, with higher education levels and income, and who had health insurance were more likely to have had visits with obstetrician-gynecologists.

**Table 3:** Adjusted associations between individual- and county-level characteristics and obstetrician-gynecologist and nurse practitioner/physician assistant visits among women age 18 to 44.

		Obstetrics-gynecology physician visits, percentage (95% CI)	*P*	Family physician visits, percentage (95% CI)	*P*	Nurse practitioner and physician, assistant visits, percentage (95% CI)	*P*
**Rural**		-2.7 (-4.9, -0.5)*	0.017	-1.0 (-4.3,2.3)	0.548	6.1 (2.7, 9.4)*	<0.001
**Individual-level characteristics**							
**Age**		0.1 (0, 0.2)*	0.004	0.3 (0.2,0.4)*	<0.001	0.1 (0, 0.1)	0.312
**Race/ ethnicity**	White, Non-Hispanic	Ref.		Ref.		Ref.	
	Black, Non-Hispanic	-1.9 (-3.6, -0.2)*	0.025	-2.5 (-4.1,-0.9)*	0.002	-6.7 (-8.2, -5.1)*	<0.001
	Other Race, Non-Hispanic	-2.3 (-4.1, -0.5)*	0.012	-3.1 (-4.8,-1.4)*	<0.001	-6.4 (-7.8, -5)*	<0.001
	Hispanic (all races)	-8.2 (-10.2, -6.2)*	<0.001	-3.4 (-5.2,-1.5)*	<0.001	-6.7 (-8.7, -4.7)*	<0.001
**Education**	Less than High School	Ref.		Ref.		Ref.	
	High School	0.7 (-0.8, 2.3)	0.352	-2.3 (-4.2,-0.4)*	0.020	1.1 (-0.4, 2.7)	0.139
	Bachelors/Graduate Degree	5.3 (3.6, 7)*	<0.001	-1.3 (-3.2,0.5)	0.162	4.3 (2.7, 5.9)*	<0.001
**Married**		4 (2.6, 5.5)*	<0.001	1.5 (0.1, 2.9)*	0.035	0.5 (-0.9, 1.9)	0.475
**Income**	Low Income	Ref.		Ref.		Ref.	
	Middle Income	1.9 (0.5, 3.2)*	0.009	0.3 (-1.18, 1.74)	0.704	1.3 (-0.1, 2.8)	0.077
	High Income	4.2 (2.6, 5.8)*	<0.001	0.3 (-1.5, 2.1)	0.736	1.7 (-0.1, 3.6)	0.069
**Health insurance**		12 (10.7, 13.2)*	<0.001	11.7 (10.2, 13.2)*	<0.001	4.8 (3.2, 6.4)*	<0.001
**Family size**		-1.4 (-1.8, -1.1)*	<0.001	-0.7 (-1.1, -0.3)*	<0.001	-1.4 (-1.8, -1.1)*	<0.001
**Employed**		1.8 (0.5, 3)*	0.005	-0.2 (-1.7, 1.2)	0.761	-0.2 (-1.6, 1.1)	0.733
**Poor or fair health status**		0.9 (-1.9, 3.7)	0.511	11.5 (8.4, 14.5)*	<0.001	3.4 (1, 5.9)*	0.006
**Poor or fair mental health status**		0.2 (-2.9, 3.4)	0.893	6.4 (3, 9.9)*	<0.001	2.7 (-0.2, 5.5)	0.067
**Pregnant**		54.3 (52.4, 56.2)*	<0.001	-3.5 (-5.5, -1.4)*	0.001	3.4 (1.5, 5.4)*	0.001
**Region**	Northeast	Ref.		Ref.		Ref.	
	Midwest	-0.7 (-3.3, 1.9)	0.598	7.3 (4.4, 10.2)*	<0.001	3.0 (-0.1, 6.1)	0.061
	South	2.3 (0, 4.6)*	0.047	2.0 (-0.4, 4.4)	0.101	-0.4 (-2.8, 2.1)	0.772
	West	-3.1 (-5.5, -0.6)*	0.014	0.3 (-2.4, 3)	0.825	3.7 (0.8, 6.6)*	0.011
**County-level characteristics**							
**County unemployment rate**		0.2 (-0.1, 0.5)	0.142	0.5 (0.2, 0.8)*	0.001	-0.5 (-0.8, -0.2)*	<0.001
**Obstetrics-gynecology physicians per 100,000 population**		0.4 (0.2, 0.5)*	<0.001	-0.4 (-0.5, -0.2)*	<0.001	-0.6 (-0.7, -0.4)*	<0.001
**Nurse practitioners and physician assistants per 100,000 population**		0 (-0.06, -0.02)*	<0.001	0 (0, 0)	0.849	0.1 (0.1, 0.1)*	<0.001
**Family medicine physicians per 100,000 population**		-0.1 (-0.1, 0)*	0.002	0.2 (0.1, 0.3)*	<0.001	0.1 (0, 0.2)*	0.002

Note: CI = Confidence Interval. Ref. = Reference group. Sample size = 37,026.

* Association between provider visits and characteristic is statistically significant at a level of p<0.05.

### Blinder-Oaxaca decomposition

Blinder-Oaxaca decomposition analysis results (Table 4) showed that, in total, only 18% (-0.59 percentage points, 95% CI = -2.38,1.19) of the total rural-urban difference (-3.28 percentage points, 95% CI = -5.81, -0.74) in obstetrician-gynecologist visits is explained by the sum total of the measured variables, leaving a sizeable residual difference. However, this total includes counterbalancing factors, some of which contribute to and others which diminish the rural-urban disparity. The lower county-level obstetrician-gynecologist supply available for rural women accounted for 2.75 percentage points (95% CI = -3.74, -1.75) of rural-urban difference, or 83.8% of the total 3.28 percentage points (95% CI = -5.81, -0.74) rural disparity. Thus, holding all other variables constant, increasing the number of obstetrician-gynecologists in rural areas would eliminate most of the rural-urban disparity in OB-GYN visits. Other factors made a smaller contribution to the disparity, including lower education level (-0.48 percentage points, 95% CI = -0.71,-0.24), lower income (-0.48 percentage points, 95% CI = -0.72,-0.23), and larger family size (-0.18 percentage points, 95% CI = -0.36,-0.01) among rural residents. Some other factors that were positively associated with OB-GYN visits mitigated the disparity and contributed to the large unexplained portion, including having predominantly white, non-Hispanic race/ethnicity (0.81 percentage points, 95% CI = 0.39,1.22), higher rates of marriage (0.25 percentage points, 95% CI = 0.09,0.41), worse health status (1.28 percentage points, 95% CI = 0.27,2.29), and lower per-capita NP/PA supply (0.75 percentage points, 95% CI = 0.23,1.27). These are factors that favored rural women and would serve to increase rural-urban disparities in obstetrician-gynecologist visits if equalized.

**Table 4:** Blinder-Oaxaca linear decomposition results for rural/urban disparities in obstetrician-gynecologist and nurse practitioner/physician assistant visits among women age 18 to 44.

	**Obstetrics-gynecology physician visits, percentage points (95% CI)**			**Family medicine physician visits, percentage points (95% CI)**			**Nurse practitioner and physician assistant visits, percentage points (95% CI)**		
**Differences**	-3.28 (-5.81,-0.74)	100%		3.40 (0.46,6.34)	100%		8.50 (5.22,11.78)	100%	
**Explained**	-0.59 (-2.38,1.19)	18%		4.41 (3.11,5.7)	130%		2.44 (0.71,4.17)	29%	
**Unexplained**	-2.69 (-4.94,-0.44)	82%		-1.01 (-3.96,1.95)	-30%		6.06 (2.82,9.29)	71%	
	**Portion of explained difference**			**Portion of explained difference**			**Portion of explained difference**		
**Measured variables**	**Absolute differences (95% CI)**	***P***	**%**^1^	**Absolute differences (95% CI)**	***P***	**%**	**Absolute differences (95% CI)**	***P***	**%**
**Individual-level characteristics**									
**Age**	-0.03 (-0.09,0.04)	0.434	0.8	-0.06 (-0.21,0.09)	0.420	-1.8	-0.01 (-0.04,0.02)	0.526	-0.1
**Race/ethnicity**^2^	0.81 (0.39,1.22)*	<0.001	-24.6	0.67 (0.33,1.02)*	<0.001	19.8	1.45 (0.97,1.93)*	<0.001	17.1
**Education**	-0.48 (-0.71,-0.24) *	<0.001	14.5	-0.03 (-0.17,0.11)	0.662	-0.9	-0.35 (-0.52,-0.17)*	<0.001	-4.1
**Marital status**	0.25 (0.09,0.41) *	0.003	-7.5	0.09 (-0.01,0.19)	0.070	2.7	0.03 (-0.05,0.12)	0.483	0.4
**Income**	-0.48 (-0.72,-0.23) *	<0.001	14.5	-0.03 (-0.24,0.18)	0.781	-0.9	-0.18 (-0.4,0.05)	0.124	-2.1
**Insurance**	-0.30 (-0.70,0.10)	0.139	9.2	-0.29 (-0.69,0.1)	0.139	-8.7	-0.12 (-0.29,0.04)	0.150	-1.4
**Family size**	-0.18 (-0.36,-0.01) *	0.042	5.5	-0.09 (-0.19,0.01)	0.068	-2.8	-0.18 (-0.36,-0.01)*	0.042	-2.1
**Employment status**	-0.06 (-0.13,0.01)	0.079	1.9	0.01 (-0.04,0.06)	0.763	0.2	0.01 (-0.04,0.05)	0.736	0.1
**Health status**	1.28 (0.27,2.29) *	0.013	-39.0	0.17 (-0.08,0.43)	0.173	5.1	0.16 (0.06,0.27)*	0.002	1.9
**Region**	0.37 (-0.12,0.87)	0.138	-11.4	1.13 (0.45,1.81)*	0.001	33.2	-0.02 (-0.56,0.53)	0.956	-0.2
**County-level characteristics**									
**County unemployment rate**	0.12 (-0.08,0.32)	0.238	-3.6	0.28 (-0.04,0.60)	0.089	8.2	-0.30 (-0.64,0.04)	0.085	-3.5
**Obstetrics-gynecology physicians per 100,000 population**	-2.75 (-3.74,-1.75) *	<0.001	83.8	2.75 (1.65,3.85)*	<0.001	80.9	4.25 (3.00,5.50)*	<0.001	50.1
**Nurse practitioners and physician assistants per 100,000 population**	0.75 (0.23,1.27) *	0.005	-22.9	0.05 (-0.46,0.56)	0.849	1.5	-2.17 (-3.15,-1.20)*	<0.001	-25.6
**Family medicine physicians per 100,000 population**	0.10 (-0.20,0.41)	0.508	-3.1	-0.24 (-0.95,0.46)	0.501	-7.1	-0.15 (-0.58,0.29)	0.508	-1.7

Note: Sample size = 37,026. Differences = Total difference between urban and rural areas. Explained = Component explained by measured variables. Unexplained = Component unexplained by measured variables.

* Contribution of characteristic to explained differences is statistically significant at a level of p<0.05.

^1^Relative percentages of total disparity.

^2^Race/ethnicity includes Non-Hispanic Whites, Non-Hispanic Blacks, Hispanics, and Non-Hispanic Other Races. Education includes Less than high school, High school, and Bachelors/graduate degree. Income includes Low income, Middle income, and High income. Region includes the U.S. Census regions of Northeast, Midwest, South, and West. Health status includes Activity limitation, Poor or fair health status, Poor or fair mental health status, and Pregnancy status.

The 3.4 percentage point (95% CI = 0.46,6.34) higher family medicine physician visit rate for rural women was entirely explained by the measured individual and county variables. In fact, rural women would have a non-significant lower rate of family medicine visits if all factors were equalized. A majority (2.75 percentage points, 95% CI = 1.65,3.85) of the observed higher rural rate is attributable to the lower obstetrician-gynecologist supply in rural areas. Higher proportions of white, non-Hispanic residents (0.67 percentage points, 95% CI = 0.33,1.02) and regional differences (1.13 percentage points, 95% CI = 0.45,1.81) also contributed to the increased likelihood for rural women having had family medicine physician visits.

Overall, roughly 29% (2.44 percentage points, 95% CI = 0.71,4.17) of the 8.5 percentage points (95% CI = 5.22,11.78) rural-urban difference in NP/PA visits was explained by available individual and county variables, in total. However, around half of the disparity (4.25 percentage points, 95% CI = 3.00,5.50) could be attributed to the lower obstetrician-gynecologist supply in rural areas. Higher proportions of white, non-Hispanic residents (1.45 percentage points, 95% CI = 0.97,1.93) and poorer health status (0.16 percentage points, 95% CI = 0.06,0.27) also contributed to the increased likelihood for rural women having had NP/PA visits, while other factors mitigated this effect, including education level (-0.35 percentage points, 95% CI = -0.52,-0.17), family size (-0.18 percentage points, 95% CI = -0.36,-0.01), and per-capita NP/PA supply (-2.17 percentage points, 95% CI = -3.15,-1.20). Decomposition analysis using a non-linear approach as a sensitivity analysis found similar results (Table 5).

**Table 5:** Fairlie non-linear decomposition results for rural/urban disparities in obstetrician-gynecologist and nurse practitioner/physician assistant visits among women age 18 to 44.

	**Obstetrics-gynecology physician visits, percentage points (%)**			**Family medicine physician visits, percentage points (%)**			**Nurse practitioner and physician assistant visits, percentage points (%)**		
**Differences**	-3.279 (100%)			3.402 (100%)			8.498 (100%)		
**Explained**	-0.864 (26%)			4.356 (128%)			3.490 (41%)		
**Unexplained**	-2.414 (74%)			-0.954 (-28%)			5.008 (59%)		
	**Portion of explained difference**			**Portion of explained difference**			**Portion of explained difference**		
**Measured variables**	**Absolute differences (95% CI)**	***P***	**%**^1^	**Absolute differences (95% CI)**	***P***	**%**	**Absolute differences (95% CI)**	***P***	**%**
**Individual-level characteristics**									
**Age**	0 (-0.01, 0.02)	0.788	-0.1	0.10 (0.06, 0.14)*	<0.001	3.0	0 (-0.01, 0.01)	0.378	-0.1
**Race/ethnicity**^2^	0.80 (0.54, 1.06)*	<0.001	-24.5	0.68 (0.42, 0.94)*	<0.001	19.9	1.78 (1.51, 2.05)*	<0.001	21.0
**Education**	-0.43 (-0.54, -0.32)*	<0.001	13.2	-0.03 (-0.14, 0.09)	0.644	-0.8	-0.28 (-0.38, -0.17)*	<0.001	-3.3
**Marital status**	0.39 (0.25, 0.53)*	<0.001	-11.9	0.17 (0.03, 0.30)*	0.017	4.9	0.08 (-0.07, 0.22)	0.301	0.9
**Income**	-0.44 (-0.63, -0.25)*	<0.001	13.4	-0.02 (-0.19, 0.15)	0.837	-0.5	-0.10 (-0.25, 0.06)	0.216	-1.1
**Insurance**	-0.15 (-0.18, -0.12)*	<0.001	4.5	-0.06 (-0.10, -0.02)*	0.001	-1.9	-0.04 (-0.06, -0.01)*	0.002	-0.4
**Family size**	-0.36 (-0.44, -0.27)*	<0.001	10.9	-0.16 (-0.23, -0.08)*	<0.001	-4.7	-0.28 (-0.36, -0.20)*	<0.001	-3.3
**Employment status**	-0.04 (-0.07, -0.01)	0.018	1.2	0 (-0.03, 0.03)	0.841	0.1	0 (-0.01, 0.01)	0.706	0.0
**Health status**	1.22 (1.15, 1.28)*	<0.001	-37.1	0.20 (0.14, 0.26)*	<0.001	5.9	0.16 (0.11, 0.22)*	<0.001	1.9
**Region**	0.19 (-0.07, 0.44)	0.150	-5.7	0.88 (0.65, 1.12)*	<0.001	26.0	0.07 (-0.17, 0.30)	0.572	0.8
**County-level characteristics**									
**County unemployment rate**	0.10 (-0.03, 0.23)	0.143	-3.0	0.26 (0.13, 0.40)*	<0.001	7.8	-0.29 (-0.43, -0.16)*	<0.001	-3.4
**Obstetrics-gynecology physicians per 100,000 population**	-2.86 (-3.56, -2.16)*	<0.001	87.2	2.79 (1.97, 3.61)*	<0.001	82.1	5.69 (4.75, 6.63)*	<0.001	67.0
**Nurse practitioners and physician assistants per 100,000 population**	0.65 (0.37, 0.92)*	<0.001	-19.7	0.06 (-0.39, 0.52)	0.784	1.9	-3.05 (-3.58, -2.52)*	<0.001	-35.9
**Family medicine physicians per 100,000 population**	0.07 (0.03, 0.11)*	<0.001	-2.1	-0.52 (-0.65, -0.40)*	<0.001	-15.4	-0.26 (-0.36, -0.16)*	<0.001	-3.1

Note: Sample size = 37,026. Differences = Total difference between urban and rural areas. Explained = Component explained by measured variables. Unexplained = Component unexplained by measured variables.

* Contribution of characteristic to explained differences is statistically significant at a level of p<0.05.

^1^Relative percentages of total disparity.

^2^Race/ethnicity includes Non-Hispanic Whites, Non-Hispanic Blacks, Hispanics, and Non-Hispanic Other Races. Education includes Less than high school, High school, and Bachelors/graduate degree. Income includes Low income, Middle income, and High income. Region includes the U.S. Census regions of Northeast, Midwest, South, and West. Health status includes Activity limitation, Poor or fair health status, Poor or fair mental health status, and Pregnancy status.

## Discussion

This study aimed to identify and better understand the rural-urban differences among women of reproductive age in having received care by the health care providers most essential for the maternal health workforce, and particularly by the birth supervising providers who are well-equipped to manage pregnancy complications and obstetric emergencies [6, 8]. Overall, there were no rural-urban difference in women having had a past-year visit with a women’s health provider or a birth supervising provider, with 65% of the sample in both urban and rural areas having had such a health visit. However, the specific types of providers seen by women varied by rural and urban geography. Rural women were found to be about 3.3 percentage points (95% CI = -5.81, -0.74) less likely than urban women to have had a past-year OB-GYN visit. Although seemingly a small difference, this potentially reflects a large number of women at the population level. Importantly, the lower rural county-level supplies of obstetrician-gynecologists accounted for the majority of this disparity, holding other covariates constant, suggesting that efforts to increase the number of obstetrician-gynecologists in rural areas would mitigate most of the observed rural disparity in OB-GYN care access. Lower obstetrician-gynecologist availability was also an influential component for why rural women were about 3.4 percentage points (95% CI = 0.46,6.34) more likely to have received care from a family medicine physician and 8.5 percentage points (95% CI = 5.22,11.78) more likely to have received care from a nurse practitioner or physician assistant. Hence, higher rates of rural women having had past-year visits with family medicine physicians, NPs, and PAs appears to largely be attributable to lower rural OB-GYN availability. Other important predictors of access to care among women of reproductive age in this study included community demographics for individual-level factors, such as race/ethnicity, education, and income levels. For example, rural-urban disparities in obstetrician-gynecologist visits observed in this study were partially explained by lower levels of education and income among rural women, while higher proportions of married and white, non-Hispanic women in rural areas served to counterbalance or mitigate disparities. This latter finding is in line with known barriers to health service access for women due to social determinants of health [20, 32]. This investigation builds upon recent findings that rural women have lower rates of past-year physician visits, and specifically obstetrician-gynecologist visits, by newly examining rural-urban differences in non-physician visits and also by identifying some of the contributing factors to the observed rural disparities in women’s health care access [17].

Although rural women in this study were less likely than urban counterparts to have had past-year visits with obstetrician-gynecologists, a positive finding was that there were no overall visit differences between rural and urban women when looking health care access for all provider types or birth supervising providers collectively. In other words, rural women of reproductive age are getting care, just by different types of providers than urban women. Primary care clinicians provide essential services for women, including comprehensive chronic disease management, counseling, and preventive care, which can be provided by both physicians and non-physicians as well as by interprofessional health care teams [4, 6, 8, 26, 27]. Although internal medicine physicians, nurse practitioners, and physician assistants can provide many of the important services that women of reproductive age and others need, referral would be needed from these providers for specialized obstetric services. Family medicine physicians and certified nurse midwives are also capable of providing obstetrical care, and the scientific literature suggests that these professionals may have key obstetric outcomes that are comparable to OB-GYNs [9–12]. The findings in this investigation are consistent with previous studies in terms of rural residents being more likely than urban counterparts to utilize nurse practitioners and physician assistants for their health care needs, and for the important role these providers serve in delivering care in rural and other areas facing primary care physician shortages [33]. And yet, a third of women from both rural and urban settings in this study did not report having a past-year visit with any of the studied provider types. ACOG notes that annual assessments by obstetrician-gynecologists and other providers offering health care to women are an excellent opportunity to provide or refer for recommended services, including screening, counseling, and preventive care [34]. Routine care visits may also help to optimize a woman’s health prior to pregnancy and facilitate early prenatal care initiation, which is important for reducing maternal and infant mortality but occurs less often for women living in rural areas [8, 35].

Improving health outcomes and addressing health disparities is central to the mission of the federal Health Resources and Services Administration, and the results of this investigation provide support for HRSA’s policy and programmatic efforts to strengthen access to a skilled health workforce in rural and other underserved areas. The type of women’s health provider from whom rural women have sought care in the past year appears to be associated with the relative availability of these providers. Hence, health workforce building may be one important strategy within multi-faceted public health initiatives aiming to combat maternal mortality and morbidity by improving access to high quality health care services [21]. While this study suggests that bolstering the rural OB-GYN workforce would eliminate of the majority of the observed disparity in OB-GYN visits for rural women, it appears that the trend of workforce sufficiency is unfortunately heading in the other direction. HRSA projects that the nation’s supply of obstetrician-gynecologists will increasingly be outpaced by the demand for them in the coming years, and that there will be disproportionate regional shortages of these physicians [13]. Solutions are needed to build up both the OB-GYN and primary care health workforce not only on a national level, but with a particular focus on the rural and other locations where physician shortages are most pronounced. Building up the health workforce in high-need rural areas tends to face a number of well-characterized challenges, such as the traditionally weaker rural labor markets that tend to hamper provider recruitment and retention [36]. However, the recent passage of P.L. 115–320 (the *Improving Access to Maternity Care Act*) amends the Public Health Service Act to require HRSA to identify “maternity care health professional target areas” within health professional shortage areas (HPSAs) that specifically have maternal health provider shortages, and to publish the data it collects data on provider availability. Such information may help to facilitate directed health workforce investments by the federal government and others toward the areas with the greatest workforce needs.

This study focused solely on health care access as it relates to office-based preventive care service delivery to women, just one component of the broad array of issues requiring action to improve maternal health and to reduce maternal morbidity and mortality [37]. Although the availability of women’s health providers appears to influence who rural women seek out for care, health workforce building is not the only solution for improving care access in rural areas. The Centers for Medicare and Medicaid Services proposes a number of other strategies which may complement this approach and could ultimately be more cost-effective, including the standardization of scope of practice laws for maternal health providers where they are currently inconsistent across states, enhancing the utilization of care coordination and home visiting services, operationalizing “hub and spoke” service models, and expanding the use of telemedicine [38]. The use of telemedicine specifically for obstetric monitoring and care delivery shows significant promise, although payment policy and lower broadband internet access in rural areas remain barriers to fully leveraging this modality [39–41]. Taken together, these strategies could be particularly important for rural communities that are unable to financially sustain OB-GYN and other provider practices in the long term. For example, variability in state scope of practice regulations for nurse practitioners and physician assistants results in inconsistent levels of autonomy for these providers in regard to their ability to make a diagnosis and develop a treatment plan, and for their prescriptive authority, despite evidence supporting the quality of the care they deliver [42, 43]. In addition, hospitals with limited obstetric services need to ensure that sufficient transfer protocols are in place or that they are part of regionalized health systems that are able to provide high quality care to pregnant women facing higher acuity health needs [19]. The extent to which rural hospitals lacking obstetric services have such transfer protocols is not known. Further, the increasing trend of rural hospital closures is concerning, along with the fact that more than half of rural counties already lack hospital-based obstetric services–particularly in the same counties where the most vulnerable patients live and that have the greatest shortages of skilled health care providers [16, 18, 21]. Hence paired with measured efforts to build up the skilled women’s health workforce, additional areas for policy focus include the prevention of rural hospital closures and the strengthening of the quality of care delivered to all pregnant women. Further investigation into the other determinant factors behind rural-urban differences in access to skilled maternal health care services may yield additional and important policy insights for addressing maternal morbidity and mortality.

The findings in this study that there were no rural-urban differences in women having had a past-year visit with at least one of the women’s health provider types studied potentially suggests that if all OB-GYNs, all family physicians, and all certified nurse midwives included pre-natal care and obstetric deliveries in their practice, that there could theoretically be an appropriately dispersed health workforce now and that there is equal potential for access to needed care for rural and urban women aged 18 to 44. This prompts a need to further investigate how the skills and services offered by these providers varies in rural and urban settings, to determine whether or not rural women are getting adequate specialized women’s health care services despite having had fewer OB-GYN visits, and to assess whether the rural delivery of care by a different cadre of providers has an effect on health outcomes. Although obstetrician-gynecologists, family medicine physicians, and certified nurse-midwives were included in this study, not all active professionals within these occupations provide obstetrical care. Hence, the county-level, per-population density of a given provider type does not necessarily translate into a measure of obstetrical care access. Although the majority (roughly 79%) of obstetrician-gynecologists are estimated to practice obstetrics, only around 5% of family medicine physicians do, with a degree of geographical variation and a notably downward trend in this practice over time [44, 45]. As such, care provision by family medicine physicians in communities without sufficiently available obstetrician-gynecologists does not directly imply that obstetrics services are available in these areas. However, rural family medicine providers are more likely to provide obstetrical care than their urban counterparts, which may be driven at least in part by relative rural shortages in obstetrician-gynecologists [45]. Declines in family medicine physicians practicing obstetrics appear to occur predominantly in areas where they provide lower volumes of deliveries, presumably due to a lesser demand for these services [44]. Notably, rural hospital obstetric unit closures occur more often in smaller hospitals and communities where there is a more limited obstetric workforce, and higher availability of practicing family medicine physicians in the community lowers the odds of an obstetric unit’s closure [46].

This study was subject to several limitations. First, a small portion of MEPS-HC respondents (6.58%) were excluded from analysis due to missing values in the individual- and county-level variables, which could potentially reduce the representativeness of the sample, or cause bias in the estimation of the parameters. Inherent differences in the values and health care expectations between individuals from rural and urban areas may affect their responses to MEPS survey questions around care access, as has been described previously, and hence may influence whether rural women seek care regardless of provider availability [17, 47]. Patient self-reported data is a theoretical limitation of all survey-based studies, although scholars generally consider such data valid for study and MEPS in particular is considered a methodologically rigorous and reliable source of health care utilization data [48, 49]. It was not possible to restrict the data available for analysis to just pregnant women or to be specific for maternal care. Thus, the results here do not identify whether or not rural women are less likely to see an obstetrician-gynecologist during pregnancy, rather just while they are of reproductive age. Data limitations in this study also prevented detailed differentiation of NPs and PAs by specialty area, such as primary care and women’s health. Around three-quarters of NPs and about 37% of PAs in the U.S. practice within these specialties [50, 51]. Despite certain limitations, this study also has a number of strengths. For one, six years of data from a nationally representative survey with a large sample size (n = 37,026) were used for analysis. In addition, while previous studies have focused on rural-urban differences in health care utilization, few studies have focused on the determinant effects of such disparities, or used an approach such as Blinder-Oaxaca decomposition analysis.

## Conclusion

There are rural-urban differences in whether women of reproductive age have received recent care by the health care providers most essential for the maternal health workforce. Rural women had lower past-year obstetrician-gynecologist visit rates than urban women, although they had higher visit rates with family medicine physicians, nurse practitioners, and physician assistants. A lower rural supply of obstetrician-gynecologists explained the largest portions of these rural-urban differences, while other individual- and county-level characteristics had smaller effects. Investments aiming to bolster the rural obstetrician-gynecologist workforce may eliminate some of the health care access disparities for rural women, and hence may be an important component of multi-pronged policy efforts aiming to mitigate maternal morbidity and mortality.
